# Therapeutic Intervention for Chronic Prostatitis/Chronic Pelvic Pain Syndrome (CP/CPPS): A Systematic Review and Meta-Analysis

**DOI:** 10.1371/journal.pone.0041941

**Published:** 2012-08-01

**Authors:** Jeffrey M. Cohen, Adam P. Fagin, Eduardo Hariton, Joshua R. Niska, Michael W. Pierce, Akira Kuriyama, Julia S. Whelan, Jeffrey L. Jackson, Jordan D. Dimitrakoff

**Affiliations:** 1 Harvard Medical School, Boston, Massachusetts, United States of America; 2 Massachusetts General Hospital, Boston, Massachusetts, United States of America; 3 Harvard School of Dental Medicine, Boston, Massachusetts, United States of America; 4 Medical College of Wisconsin, Milwaukee, Wisconsin, United States of America; 5 Kurashiki Central Hospital, Okayama, Japan; The James Cook University Hospital, United Kingdom

## Abstract

**Background:**

Chronic prostatitis/chronic pelvic pain syndrome (CP/CPPS) has been treated with several different interventions with limited success. This meta-analysis aims to review all trials reporting on therapeutic intervention for CP/CPPS using the National Institutes of Health-Chronic Prostatitis Symptom Index (NIH-CPSI).

**Methods:**

We searched Medline, PubMed, the Cochrane Pain, Palliative & Supportive Care Trials, the Cochrane Register of Controlled Trials, CINAHL, ClinicalTrials.gov, and the NIDDK website between 1947 and December 31, 2011 without language or study type restrictions. All RCTs for CP/CPPS lasting at least 6 weeks, with a minimum of 10 participants per arm, and using the NIH-CPSI score, the criterion standard for CP/CPPS, as an outcome measure were included. Data was extracted from each study by two independent reviewers. Gillbraith and I-squared plots were used for heterogeneity testing and Eggers and Peters methods for publication bias. Quality was assessed using a component approach and meta-regression was used to analyze sources of heterogeneity.

**Results:**

Mepartricin, percutaneous tibial nerve stimulation (PTNS), and triple therapy comprised of doxazosin + ibuprofen + thiocolchicoside (DIT) resulted in clinically and statistically significant reduction in NIH-CPSI total score. The same agents and aerobic exercise resulted in clinically and statistically significant NIH-CPSI pain domain score reduction. Acupuncture, DIT, and PTNS were found to produce statistically and clinically significant reductions in the NIH-CPSI voiding domain. A statistically significant placebo effect was found for all outcomes and time analysis showed that efficacy of all treatments increased over time. Alpha-blockers, antibiotics, and combinations of the two failed to show statistically or clinically significant NIH-CPSI reductions.

**Conclusion:**

Results from this meta-analysis reflect our current inability to effectively manage CP/CPPS. Clinicians and researchers must consider placebo effect and treatment efficacy over time and design studies creatively so we can more fully elucidate the etiology and role of therapeutic intervention in CP/CPPS.

## Introduction

Chronic prostatitis/chronic pelvic pain syndrome (CP/CPPS) is defined as “urologic pain or discomfort in the pelvic region, associated with urinary symptoms and/or sexual dysfunction, lasting for at least 3 of the previous 6 months” in the absence of any identifiable pathology such as cancer, culturable infection, or anatomic abnormalities, often accompanied by “associated negative cognitive, behavourial, sexual or emotional consequences.”[1], [2] CP/CPPS is a heterogeneous condition with broad diagnostic criteria, a lack of any validated biomarkers, and many possible etiologies that share the same symptomatic end point.[3] The heterogeneity of CP/CPPS and the current inability of the medical community to reliably identify the subgroups of this disease have made finding effective treatment regimens challenging.

Our study purpose is to assess which treatment modalities are effective in treating CP/CPPS by synthesizing the data from all randomized controlled trials (RCTs) for CP/CPPS since 1999. Nineteen ninety-nine is the year that the National Institutes of Health-Chronic Prostatitis Symptom Index (NIH-CPSI) was validated. This instrument is a widely accepted graded uniform outcome measure that standardizes measurement of CP/CPPS symptoms, allowing more accurate comparisons between studies. The self-administered questionnaire is highly discriminative for CP/CPPS, focusing on the location, severity, and quality of pain, irritative and obstructive urinary function, and patients’ overall quality of life.[4] With this aggregate data, we hope to obtain enough power to provide a statistically significant and clinically meaningful analysis that could provide treatment insights to practicing clinicians.

## Methods

### Searching

This report employs the PRISMA statement for reporting systematic reviews.[5] We searched Medline and PubMed (1947 - December 31, 2011) using a search strategy designed by a medical librarian (JW) and presented in the Supplementary Online Information section without restrictions on language or study type. In addition, we searched EMBASE, CINAHL, PsycInfo, Alt HealthWatch Online, the Cochrane Registry of Controlled Clinical Trials, Web of Science, BIOSIS Previews, ProQuest Dissertations and Theses PQDT, and Factiva (see Table S1). We utilized the NLM Gateway Meeting Abstracts and Conference Papers Index to capture meeting abstracts. We looked for additional clinical trial listings in Cochrane Pain, Palliative & Supportive Care Trials Register, the Cochrane Central Register of Controlled Trials, ClinicalTrials.gov, Cochrane Trial Registry, mRCT, CenterWatch, and pharmaceutical company web sites. We searched Google Scholar and the National Institute of Diabetes and Digestive and Kidney Diseases websites for grey literature. Finally, we reviewed the bibliographies of all articles retrieved. The last search was performed December 31, 2011.

### Selection and Validity Assessment

Inclusion criteria for retrieved studies included: 1) randomized controlled trials (either placebo or comparative effectiveness trials), 2) trials evaluated exclusively treatments of chronic prostatitis/chronic pelvic pain syndrome (NIH Category III Prostatitis), [1] 3) were at least six weeks in duration, 4) included at least 10 individuals per arm, and 5) utilized the NIH-CPSI, a graded uniform outcome measure of pain, urinary function and quality of life. We excluded trials that examined treatments for other prostatitis syndromes (NIH Categories I, II, and IV).

**Figure 1:**
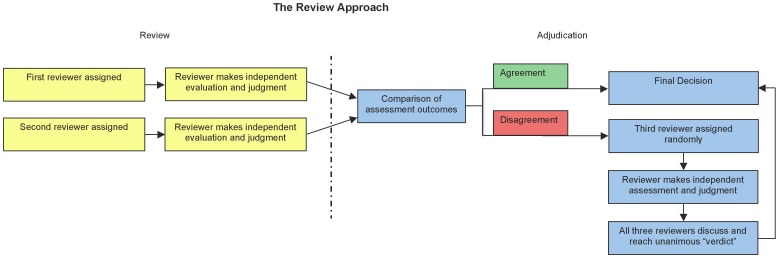
Reviewer Adjudication Strategy.

Article titles and abstracts were initially reviewed by two independent authors to determine eligibility for inclusion (Figure 1). Full text was reviewed when deemed necessary and if available. Each author independently determined whether a given paper should move to the next round, and the two authors’ opinions were compared. If the reviewers agreed, the decision was final. If the reviewers disagreed, a third reviewer discussed the trial with the two initial reviewers, and unanimous agreement was reached.

### Data Abstraction and Study Characteristics

Two authors extracted data, including study characteristics (country of origin and language), information about the intervention (design, inclusion criteria, treatment characteristics, dose and duration), subject characteristics (age), and treatment outcomes (NIH-CPSI scores and adverse events). Abstraction was done independently but not blindly. For continuous outcomes based on the NIH-CPSI, we abstracted the mean and variance of reported domains and the time point at which the data was collected. Missing variances were imputed from reported p-values.[6], [7] For dichotomous outcomes, based on significant clinical improvement as defined by each study, we abstracted data into 2×2 tables. We assessed articles using both the Jadad Scale and the Cochrane Risk of Bias Assessment.[8], [9]

### Quantitative Data Synthesis

Data were pooled using the DerSimonian and Laird random effects model using p<0.01 as our threshold for significance based on the large number of analyses.[7], [10] For studies with more than one arm, we combined arms by pooling the data into a single arm as recommended by the Cochrane Collaboration.[11] Galbraith plots and I-square were used as visual models for assessing heterogeneity.[12], [13] We tested for publication bias using the methods of Egger (for continuous outcomes) and Peter (dichotomous outcomes).[7], [14], [15] We used stratified analysis and meta-regression to identify and analyze possible sources of heterogeneity.[16] We also used a regression analysis to stratify studies by inclusion criteria. Meta-regression was performed using random effects maximum likelihood ratios, with the proportion of between-study variance explained using the Knapp-Hartung modification.[17] Planned analyses included whether or not the study included intention to treat, patient average age, trial size, trial duration, percentage of dropouts, placebo effect, and quality. For quality, we used a components approach, in which each quality measure from both JADAD and the Cochrane Risk of Bias instrument were assessed (i.e. appropriateness of randomization, appropriateness of blinding) for potential impact on our outcomes. To determine the placebo effect, we calculated a weighted mean difference, comparing the outcome for the placebo arm between baseline and subsequent time points. To evaluate the effect of time on our outcomes, we conducted meta-regression using the time point at which the data was reported as a covariate, adjusting for clustering by study. All analyses were done using STATA (v 12.0, College Station, TX). There was no external funding for this study.

## Results

### Trial Flow/Flow of Included Studies

**Figure 2:**
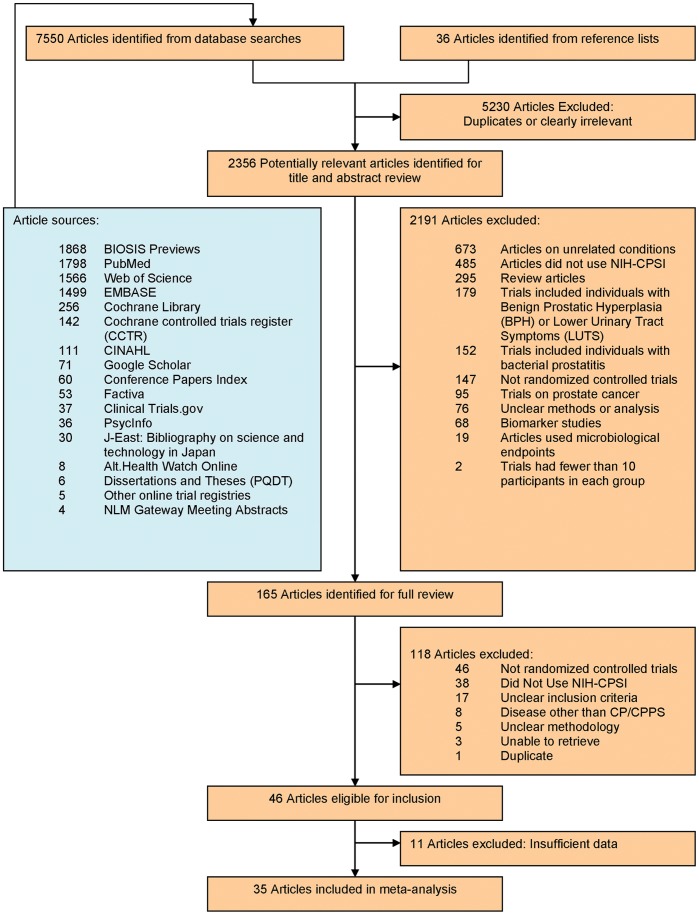
Study Selection Strategy.

Our search strategy returned 7550 potential articles (Figure 2, Figure S1, Figure S2 and Table S2). Application of our inclusion and exclusion criteria yielded 46 articles. Further review resulted in 11 additional exclusions due to insufficient data, leaving 35 articles for the study.[18]–[52] Among these 35 RCTs, 20 included a placebo (n = 16) [18]–[33] or a sham control (n = 4) [34]–[37], while 15 [38]–[52] compared different modalities or combinations of treatments directly (Table S2).

### Study Characteristics

Placebo-controlled RCTs averaged 13.4 weeks in duration (95% CI: 8.9–17.9, range: 6–52) and subjects averaged 41.1 years (95% CI: 38.4–43.8). Three classes of medications were compared to placebo in more than one trial: alpha-blockers (n = 8),[18]–[25] antibiotics (n = 2),[18], [26] and non-steroidal anti-inflammatory medications (NSAIDs) (n = 2).[30], [31] Many different medications were compared to placebo in single trials: finasteride,[27] the glycosaminoglycan pentosan polysulfate,[28] mepartricin,[29] *Secale cereale* pollen extract,[32] and pregabalin.[33] Interventions compared to sham in single trials included: acupuncture,[34] aerobic exercise,[35] extracorporeal shock wave therapy (ESWT),[36] and percutaneous posterior tibial nerve stimulation (PTNS).[37] Sixteen of these placebo-controlled trials (80%) used intention to treat analysis.[18], [22], [23], [25]–[37] Fourteen (70%) described an adequate sequence generation[18], [19], [22]–[24], [26]–[34] and all but two trials[29], [37] were adequately blinded.

### Quantitative Data Synthesis

Fifteen trials analyzed direct comparisons of various therapies or different dosage of the same therapies (Table S2).[38]–[52] Three of these trials compared antibiotics to alpha-blockers.[18], [39], [48] Five studies compared antibiotics to a combination of alpha-blockers and antibiotics.[18], [39], [40], [48], [49]

Of the 35 trials analyzed, the average Jadad score was 5.4 out of a possible eight points, with a median of 5 points; the range was two to eight (Table S3).[8] While all 35 studies were randomized, only 14[18], [22]–[24], [26], [29]–[34], [43]–[45] were categorized as randomized appropriately on the Jadad system (Table S3). Of the 35 randomized studies, 21[18]–[24], [26]–[37], [44], [50] were blinded and 19[18]–[24], [26], [28], [30]–[37], [44], [50] were blinded appropriately. All studies analyzed included descriptions of the statistical methods used and the inclusion and exclusion criteria for study participants (Table S3). Eight of the trials were sponsored by industry,[19]–[21], [26]–[28], [30], [32] six were clearly not sponsored by industry,[18], [22], [29], [33], [36], [48] and for the remaining 21 it was unclear in the text (Table S4)[23]–[25], [31], [34], [35], [37]–[47], [49]–[52].

### Placebo-Controlled Trials

#### NIH-CPSI total score

**Figure 3:**
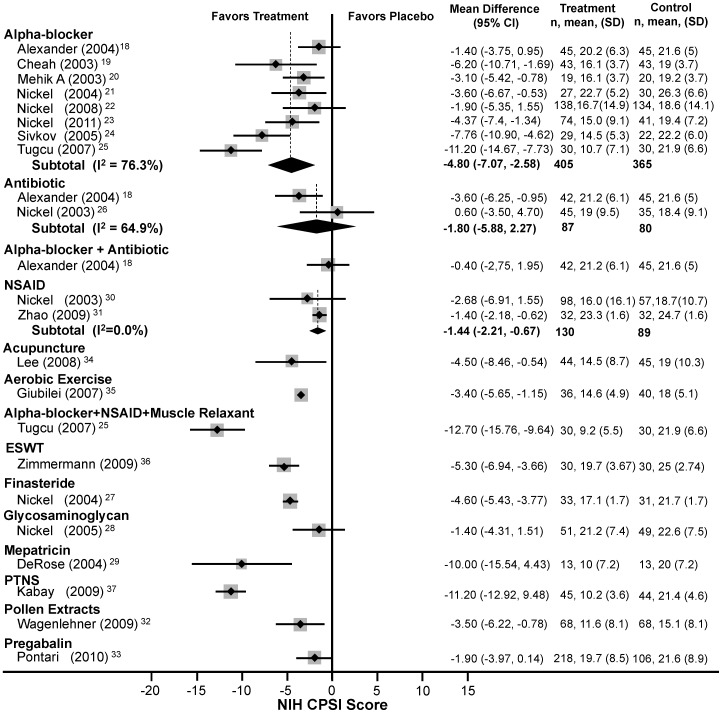
Forest Plot of Changes in the total NIH-CPSI Score.

The most frequently studied modality was alpha-blockers (Figure 3). Among eight RCTs (n = 770)[18]–[25] comparing alpha-blockers to placebo, an average total NIH-CPSI score reduction of 4.8 (95% CI: −7.1 to −2.6) was observed with high heterogeneity (Q = 29.49, df = 7, p<0.0005, I^2^ = 76.3%). Neither antibiotics (n = 167)[18], [26] nor NSAIDs (n = 219)[30], [31] resulted in significant improvement in total NIH-CPSI compared to placebo (WMD: −1.8, 95% CI: −5.9 to 2.3 for antibiotics and WMD: −1.4, 95% CI: −2.2 to −0.7 for NSAIDs). Results showed modest heterogeneity for antibiotics (Q = 2.85, df = 1, p<0.0005, I^2^ = 64.9%) and low heterogeneity for NSAIDs (Q = 0.34, df = 1, p = 0.56, I^2^ = 0.0%). Meta-regression and sensitivity analyses (see below) failed to identify the source of heterogeneity.

Single trials with mepartricin (mean difference: –10.0, 95% CI: −15.5 to 4.4)[29] and PTNS (mean difference: −11.2, 95% CI: −12.9 to 9.5)[37] had a statistically significant average improvement of six points or greater in the NIH-CPSI total score, considered clinically significant by the Chronic Prostatitis Collaborative Research Network (CPCRN).[53] In another single trial, the triple combination of doxazosin + ibuprofen + thiocolchicoside (DIT) also significantly reduced NIH-CPSI total score both clinically and statistically (mean difference: −12.7, 95% CI: −15.8 to −9.6).[25] Other therapies provided statistically significant but clinically insignificant improvement in single trials as follows: finasteride (mean difference: −4.6, 95% CI: −5.4 to −3.8),[27] *Secale cereale* (Cernilton), a proprietary rye pollen extract (mean difference: −3.5, 95% CI: −6.2 to −0.8),[32] acupuncture (mean difference: −4.5, 95% CI: −8.5 to −0.5),[34] aerobic exercise (mean difference: −3.4, 95% CI: −5.7 to −1.2),[35] and ESWT (mean difference: −5.3, 95% CI: −6.9 to −3.7).[36] Glycosaminoglycan (pentosan polysulfate, PPS),[28] NSAIDs,[30], [31] and pregabalin[33] did not significantly improve NIH-CPSI total scores, either statistically or clinically.

#### NIH-CPSI pain domain subscore

**Figure 4:**
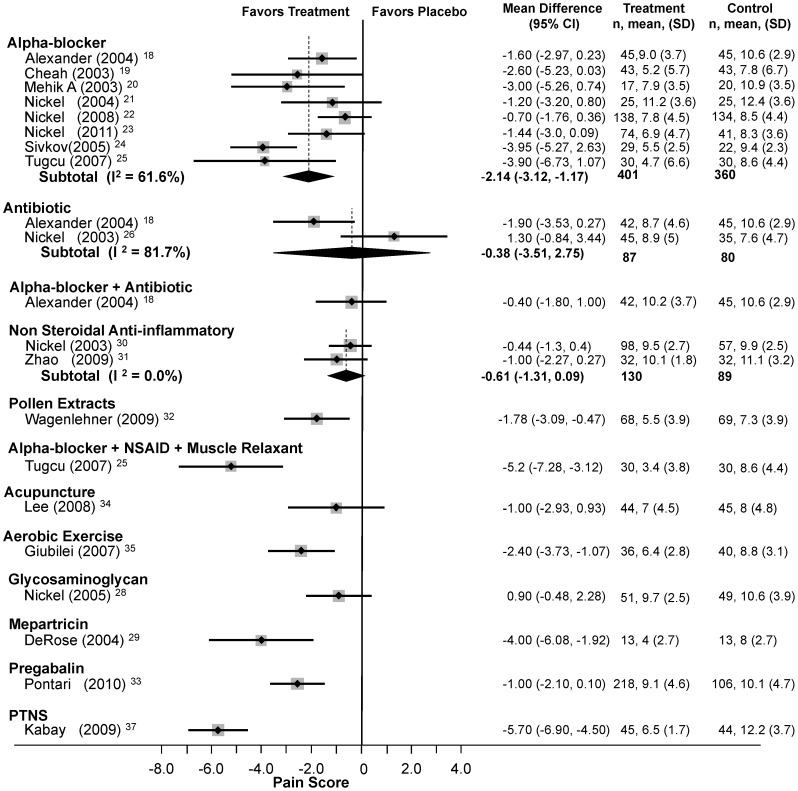
Forest Plot of Changes in the NIH-CPSI Pain Domain Score.

Eight trials (n = 761)[18]–[24] compared alpha-blockers to placebo (Figure 4), reporting an average pain reduction of 2.1 points (95% CI: −3.1 to −1.2) with moderate heterogeneity (Q = 18.24, df = 7, p = 0.01, I^2^ = 61.6%). Two trials (n = 167)[18], [26] studied antibiotics with no significant effect (WMD: −0.38, 95% CI: −3.5 to 2.8) but with high heterogeneity (Q = 5.45, df = 1, p<0.02, I^2^ = 81.7%). Two other trials (n = 219)[30], [31] demonstrated lack of efficacy of NSAIDs (WMD: −0.61, 95% CI: −1.3 to 0.1) with low heterogeneity (Q = 0.34, df = 1, I2 = 0.0%). Meta-regression and sensitivity analyses (see below) failed to identify the source of heterogeneity.

In single trials (Figure 2), *Secale cereale* pollen extract (mean difference: −1.78, 95% CI: −3.09 to −0.47),[32] combination DIT (mean difference: −5.2, 95% CI: −7.3 to −3.1)[25], aerobic exercise (mean difference: −2.4, 95% CI: −3.7 to −1.1),[35] mepartricin (mean difference: −4.0, 95% CI: −6.1 to −1.9),[29] and PTNS (mean difference: −5.7, 95% CI: −6.9 to −4.5)[37] significantly reduced the NIH CPSI pain domain subscore. In the remaining trials alpha-blockers plus antibiotics,[18] acupuncture,[34] glycosaminoglycan,[28] and pregabalin[33] did not significantly improve pain scores.

#### NIH-CPSI voiding domain subscore

Seven trials (n = 724)[18], [19], [21]–[24] compared alpha-blocker to placebo and found an average reduction of 1.1 points in the NIH-CPSI voiding domain subscore (95% CI: −1.7 to −0.4) with moderate heterogeneity (Q = 17.7, df = 6, p = 0.007, I^2^ = 66.1%). Two trials (n = 167)[18], [26] studied antibiotics with no significant effect (WMD: −0.04, 95% CI: −0.7 to 0.6) and low heterogeneity (Q = 0.07, df = 1, p = 0.80, I^2^ = 0.0%). Meta-regression and sensitivity analyses (see below) failed to identify the source of heterogeneity.

**Figure 5:**
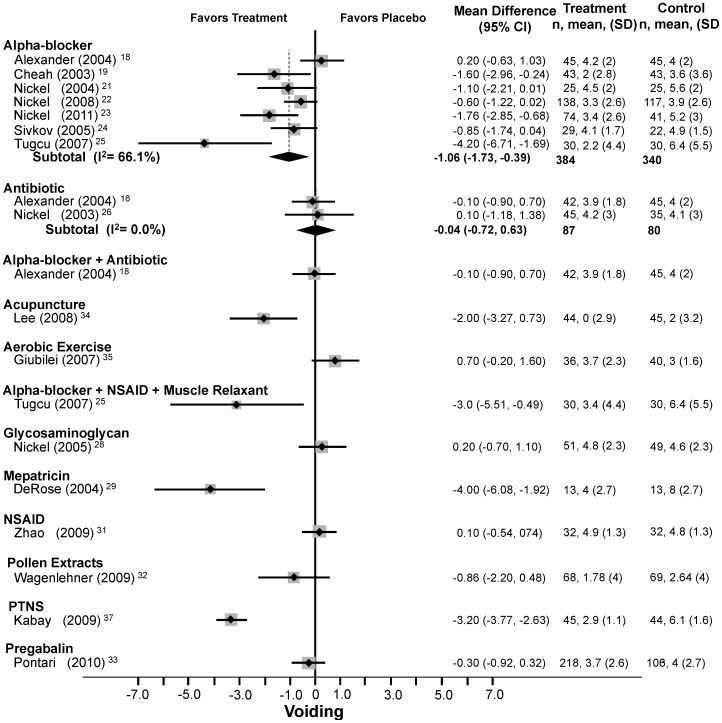
Forest Plot of Changes in the NIH-CPSI Voiding Score.

In single trials (Figure 5), combination DIT (mean difference: −3.0, 95% CI: −5.5 to −0.5),[25] *Secale cereale* pollen extract (mean difference: −0.9, 95% CI: −2.2 to −0.5),[32] acupuncture (mean difference: −2.0, 95% CI: −3.3 to −0.7),[34] and PTNS (mean difference: −3.2, 95% CI: −3.8 to −2.6)[37] significantly reduced the NIH-CPSI voiding domain subscore. In single trials, the combination of an alpha-blocker and an antibiotic,[18] glycosaminoglycan,[28] mepartricin,[29] NSAIDs,[30], [31] pregabalin[33] and aerobic exercise[35] did not improve voiding.

#### NIH-CPSI quality of life domain subscore

Seven trials (n = 770)[18], [19], [21]–[24] compared alpha-blockers to placebo and found an average reduction of 1.4 points (95% CI: −2.3 to −0.4) with high heterogeneity (Q = 36.8, df = 6, p<0.0005, I^2^ = 83.7%). Two trials (n = 167)[18], [26] studied antibiotics with no significant improvement (mean difference; −0.7, 95% CI: −1.9 to 0.5) and high heterogeneity (Q = 13.8, df = 2, p = 0.01, I^2^ = 76.3%). Meta-regression and sensitivity analyses (see below) failed to identify the source of heterogeneity.

**Figure 6:**
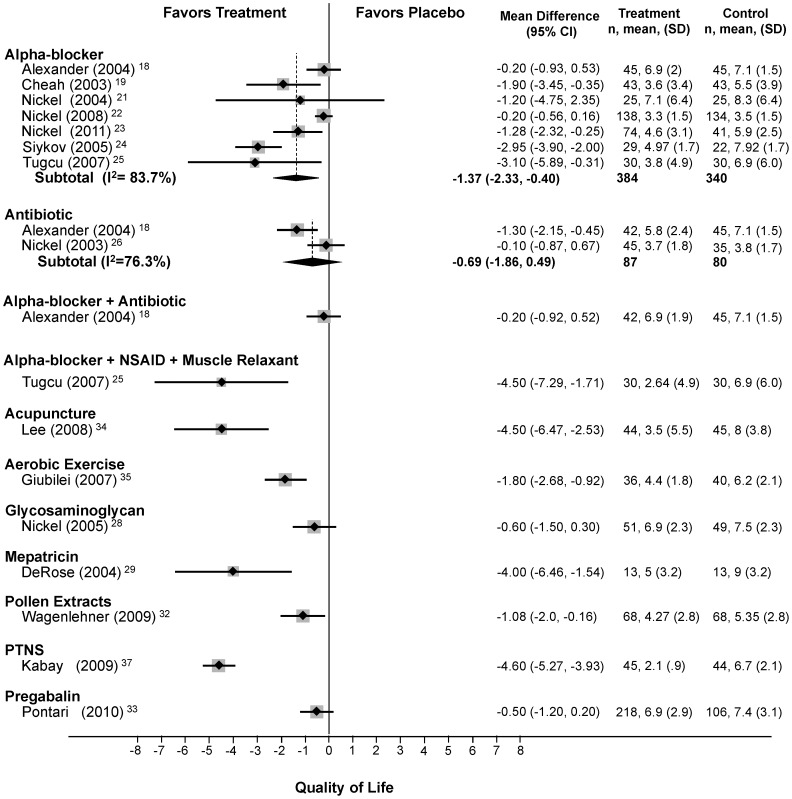
Forest Plot of Changes in the NIH-CPSI QoL Score.

In single trials, combination DIT (mean difference: −4.5, 95% CI: −7.3 to −1.7),[25] mepartricin (mean difference: −4.0, 95% CI: −6.5 to −1.5),[29] *Secale cereale* pollen extract (mean difference: −1.08, 95% CI: −2.0 to −0.16),[32] acupuncture (mean difference: −4.5, 95% CI: −6.5 to −2.5),[34] aerobic exercise (mean difference: −1.8, 95% CI: −2.7 to −0.9),[35] and PTNS (mean difference: −4.6, 95% CI: −5.3 to −3.9)[37] significantly improved the NIH-CPSI quality of life (QoL) domain subscore (Figure 6). In single trials, the combination of an alpha-blocker and an antibiotic,[18] glycosaminoglycan (PPS)[28] and pregabalin[33] did not improve voiding.

### Global Improvement

Patients treated with alpha-blockers failed to report improvement in their symptoms more frequently than patients treated with placebo (RR: 1.1, 95% CI: 0.86–1.39), moderate heterogeneity (Q = 13.5, df = 6, p<0.005, I^2^ = 55.7%).[18], [20]–[23] One study of antibiotics found no global improvement (RR: 1.0, 95% CI: 0.48–2.09).[18] Single studies that found no improvement included finasteride (RR: 2.3, 95% CI: 0.8–6.7)[27] and pregabalin (RR: 1.3, 95% CI: 1.0–1.8).[33] In single trials, PPS (RR: 2.1, 95% CI: 1.0–4.28),[28] ESWT (RR: 27, 95% CI: 1.7–4.35),[36] and PTNS (RR: 36.2, 95% CI: 2.2–5.83)[37] were effective in providing global improvement. Patients given *Secale cereale* pollen extracts were less likely to improve symptoms than those given placebo (RR: 0.7, 95% CI: 0.5–0.95).[32]

### Placebo Effect

There was a statistically significant placebo effect for all outcomes. Total NIH-CPSI score improved on average 2.4 points (95% CI: 1.7–3.2). There was a significant placebo effect for all subdomains as well: pain: 1.34 (95% CI: 0.88–1.79); voiding: 0.59 (95% CI: 0.33–0.84); quality of life: 0.95 (95% CI: 0.62–1.27). There was no evidence of changing placebo effect over time (β = 0.10, 95% CI: −0.10–0.31).

### Time Analysis

The efficacy of treatment for all modalities increased over time (β = 0.19, 95% CI: 0.11–0.27). For every additional week of any given treatment, total NIH-CPSI score decreased by an average of 0.19 points. This would imply that 32 weeks of treatment would be required to achieve a total NIH-CPSI reduction of 6 points. Alpha-blockers were the only specific class of treatment for which there was sufficient number of trials to perform a time analysis apart from other treatments. For all domains of the NIH-CPSI total score, alpha-blockers showed significant evidence of improved efficacy with longer treatment durations (Table S5).

### Direct Comparisons of CP/CPPS Treatments

Only alpha-blockers and antibiotics had sufficient number of trials to compare their effectiveness for CP/CPPS. Five trials compared alpha-blockers and antibiotics as well as combinations of the two (Table S6).[18], [39], [40], [48], [49] In direct comparison, no statistically significant difference was observed between antibiotics and alpha-blockers in their impact on NIH-CPSI total and subscores (Table S7). Combining an alpha-blocker with an antibiotic also failed to produce greater NIH-CPSI total or subscore improvement than either modality alone. All other direct comparisons involved single trials only, creating difficulty when trying to arrive at definitive conclusions (Table S6).

### Sensitivity Analyses

We did sensitivity analyses for all studies and the subgroup of alpha-blocker trials since they were the only subgroup with sufficient numbers (Table S8). We found no evidence of publication bias for either all studies or the alpha-blocker trials for the NIH-CPSI total scores (overall: p = 0.36, alpha: p = 0.11), pain (overall: p = 0.17, alpha: p = 0.22), voiding (overall: p = 0.44, alpha p = 0.28), or quality of life (overall: p = 0.07, alpha p = 0.92). For all studies, older age was associated with worse outcomes, longer study duration with better outcome (see above), studies that required CPCRN definitions, a specific NIH-CPSI score for entry or subjective symptoms for study entry had worse outcomes than less rigorous studies. Among quality markers, inadequate sequence generation and lack of concealed allocation explained some of the heterogeneity (Table S8). This combination of variables explained 95% of the between-study heterogeneity, though even with these variables, leaving minimal residual heterogeneity (I^2^ = 30.4%). For the alpha-blocker trials, study duration and requiring an NIH CPSI cut-off score for study entry both explained all the between study heterogeneity.

## Discussion

This systematic review and meta-analysis shows that many treatments for CP/CPPS are largely ineffective. Our results demonstrate several critical issues underlying the limited success of clinical trials in CP/CPPS, including the roles of placebo and treatment duration.

Single trials of mepartricin,[29] PTNS[37] and combination DIT[25] showed at least a six-point overall reduction in total NIH-CPSI score. Several methodological limitations preclude the generalizability of those findings, namely: inadequate blinding and the subsequent allocation bias (De Rose *et al.* [29]), imperfect allocation concealment (Kabay *et al*.[37] and Tuğcu *et al.* [25]), inappropriate randomization, inadequate withdrawal reporting, and lack of detailed reporting of adverse effects (Kabay *et al*.[37]). Most concerning in the Kabay *et al*.[37] study was the fact that while the treatment group received the maximum tolerable electrical stimulation, the “sham group” did not meet the definition of a sham since they did not receive any stimulation and were not blinded, prompting concerns that participants were able to differentiate between treatment and “sham.” This may be reflected in both in the efficacy of the treatment group as well as nearly absent “placebo effect” in the control group.

The alpha blocker trials included in the present meta-analysis demonstrated a high degree of heterogeneity (I^2^ = 76.3% for total NIH-CPSI and I^2^ = 61.6% for the NIH-CPSI Pain Subscore). We explored several sources of this heterogeneity which might have introduced bias in the design, execution and subsequent interpretation of those RCTs.

First, we examined the variability in the rigorousness of application of the eligibility criteria used for patient selection at randomization. We distinguished three levels of sensitivity and rigor of definition: CPCRN criteria, NIH criteria and “unclear”. For example, some studies explicitly stated that they employed the CPCRN criteria.[18], [21]–[23], [33] Other studies[20], [31], [54] referred to the JAMA 1999 Krieger criteria[1] as “the NIH criteria” which, although similar to the CPCRN criteria, do not specify conditions exclusionary to the diagnosis of CP/CPPS and are, therefore, less rigorous. Several studies did not clearly describe the criteria used for defining the patient population.[37], [42], [52] Studies that failed to use CPCRN or NIH criteria reported greater effectiveness of treatment.[37], [42], [48], [50], [52]

Secondly, we examined the importance of patient age. Generally, it has been accepted that CP/CPPS patients younger than 50 might respond differently to alpha-blocker treatment than those older than 50, mostly as a result of biological variables, such as the increased prevalence of BPH in the older patient population. Our analysis found that older patients had less response to CP/CPPS symptoms than younger patients, a paradoxical finding. This finding should be interpreted with caution for several reasons. First, this analysis is based on average age among all participants in each specific study; fully elucidating age effects on treatment response would require patient-level data. Secondly, the average age of participants in each study was constrained and relatively young, ranging from 29 to 50 years in age. Benign prostatic hyperplasia is not a significant problem in men of this age, reducing potential benefit from alpha-blockers on BPH. Given these limitations, at least four possible explanations can be evoked for this finding: 1) Longer symptom duration in older patients prior to treatment initiation; or 2) Older patients preferentially enrolled in trials of shorter duration; or 3) Older patients that were already treatment refractory at study initiation; or 4) Publication bias. While our analysis did not reveal any preferential enrollment based on age (thus, arguing against the first explanation) or treatment refractoriness at baseline (thus, arguing against the second one as well), our analysis might have been limited by the lack of detail in terms of age or treatment-based enrollment status. We did not detect any age-based publication bias either. Thus, the most plausible explanation appears to relate to the fact that longer duration of symptoms in older patients might explain the paradoxical less favorable response to treatment. Our findings are in line with the Turner study in which men with a first lifetime episode of prostatitis/pelvic pain syndrome had better outcomes compared with men with a recurrent episode.[55] The best and only available review on the natural history of CP/CPPS comes from Kusek and Nyberg from the NIH/NIDDK.[56] They observed that “among nearly 300 men with longstanding CP/CPPS (mean 6.8 yr since diagnosis) recruited from tertiary care centers and followed for 2 yr in the CPCRN study, 45% reported that they were markedly or moderately improved on the Global Response Assessment. Importantly, there was no evidence of clinically significant progression of symptoms. These investigators, however, failed to identify baseline demographic or clinical studies which predicted improvement.”[56]. In addition, the same authors reference the Nickel *et al.* studies[57], [58] reporting of all men in the community identified to have prostatitis-like symptoms, approximately one-third did not report these symptoms one year later. The preceding studies, as well as our finding of age-related paradoxical treatment response, related to the phenomenon of “prostatitis burning itself out” and a decrease in symptom severity with longer duration, thus causing a greater degree of regression to the mean in older patients. Future epidemiologic studies should provide further insight into mechanisms underlying this intriguing finding.

In addition, it has been hypothesized that alpha-blocker-naïve patients might respond differently (better) to alpha-blockade than those with previous alpha-blocker exposure. Some trials explicitly stated that “patients were excluded if they had ever previously taken alpha-blockers”[19], [22], [23], while others stated that patients were excluded only if they had taken an alpha-blocker within a pre-specified period of time before study enrollment.[18] We believe this distinction might be important and might give rise to substantial heterogeneity in effect size since the alpha-blocker-naïve population might be different from the “alpha-blocker washout” population. We found no impact of pre-treatment with alpha-blockers, either for all studies or for those studies focusing on alpha-blocker treatment.

Other important variables included duration of study and a number of quality markers including lack of adequate sequence generation and allocation concealment. Weaker studies reported greater effectiveness with treatment. This has been seen previously in systematic reviews and including weaker trials in analyses may overestimate potential benefit. In our study, we found little benefit.

Our analysis raises the issue of placebo effect in CP/CPPS clinical trials. The improvement for the pool of all placebo groups is significant for CP/CPPS symptoms overall and for all three NIH-CPSI domains. For pain, such findings are consistent with a larger body of literature that has shown contextual elements of treatment to have the most powerful effects specifically in analgesia. Without a no-treatment comparison group, however, the placebo groups in these studies also capture the improvement of symptoms due to the natural history of an unstable course of illness. The lack of change in improvement over time for the placebo groups, however, weakens natural history as a sole explanation for these results. Furthermore, previous studies of CP/CPPS have suggested placebo effect can be significant, at least over the short term, for up to three months. The placebo-controlled studies in the present meta-analysis, with an average length of 13.4 weeks, are not of sufficient length to conclude whether placebo effect might wane in the long term.

While double-blind RCTs have long been the “gold standard” for limiting bias in clinical medicine, they are not without their own limitations and biases.[59] Their underlying assumption is that by blinding both provider and patient, the placebo effect arising should be identical in both groups and, therefore, impact findings from both groups equally. However, this assumption has been challenged. One study in particular found a clinically significant difference between albuterol treatment and three control treatments (two placebos and no treatment) when patients were evaluated using a “hard” outcome variable, FEV1.[60] Surprisingly, the clinical significance between the interventions evaporated when looking at a “soft” outcome variable, a subjective patient response questionnaire. This suggests that for “soft” outcome variables the placebo effect in the control group is greater than the placebo effect in the treatment group, thereby undermining an underlying assumption of double-blind RCTs.[60] All studies included in this meta-analysis use a “soft” primary outcome variable, the NIH-CPSI score. If a similar phenomenon occurred in these studies and the placebo effect is more substantial in control than treatment groups, then the treatment effect could be blunted.

Given these considerations, it appears that there was a significant placebo effect captured in the studies included in this analysis. This itself is noteworthy because it suggests that contextual elements of care play a measurable role in patient improvement for a condition not easily treated. Patients with a chronic pain condition have often tried many failed treatments, a history of which could in turn produce negative expectations for new therapies. Therefore, why a placebo effect exists for CP/CPPS warrants further investigation because expectancy of pain relief is one primary mechanism of placebo analgesia. Qualitative research with participants in CP/CPPS trials may be the most effective manner to gain such an understanding.

### Limitations

There are several important limitations to our review. First, for nearly all modalities demonstrating efficacy, there were only small, single-center trials. Particularly for a syndrome with such a powerful placebo effect, replication and confirmation of efficacy needs to be done before any modality can be conclusively stated to be helpful. Second, there was a wide range of study quality. Several trials had questionable placebo groups and inadequate blinding. This makes interpretation of the results difficult and may lead to erroneous conclusions. This reiterates the importance of replication and confirmation. Third, there may be specific patient subsets, which might respond to specific medications. The heterogeneity of CPPS makes identifying this subgroup difficult. The “therapeutic efficacy” of certain medications might be “diluted” because responders may be overwhelmed by non-responders.[61] Lack of patient-level data made it difficult for us to fully explore such potential subgroups. Fourth, most of the studies were relatively short, averaging only 13.4 weeks in duration. While our analysis suggested continued improvement over time, the longest studies were 32 weeks; it is impossible to speculate what might happen with longer studies. Patients could continue to improve, they could plateau or they could worsen. Longer studies are needed. Finally, we were not able to analyze based on specific, important patient characteristics such as disease duration or inclusion of treatment refractory versus treatment naïve patients. This would require patient-level data or the analysis to be by the authors in the individual manuscripts. It would be nice for the authors to make an assessment of the effect of disease duration in patients (<2 yrs versus >2 yrs) and inclusion of treatment refractory versus treatment naive participants on the effectiveness of the various therapies. It is possible that patients with longer duration or previously refractory treatment would be less responsive than those with shorter disease duration or treatment naïve.

Our study also shows the potential importance of treatment duration. In particular, the pooled data for alpha-blockers showed increased efficacy over time in addition to statistically significant improvements for overall NIH-CPSI and all three subdomains. Given the relatively short average study length and the 4.5 point average reduction in total NIH-CPSI for alpha-blockers, longer studies of alpha-blockers might result in clinically significant reductions in NIH-CPSI total score if the trend continues over time. For the pooled treatment data, the NIH-CPSI score decreased an average of 0.23 points each week, suggesting that 26 weeks of treatment would be required to see a six point reduction in the total NIH-CPSI score. Our study has significant difference from a recently published network meta-analysis (Table S9).[62]

### Conclusion

The mixed results of the studies in this meta-analysis highlight the heterogeneity of CP/CPPS and our current lack of understanding of the etiology of the disease. Furthermore, our results highlight the significant limitations of previous trials, the existence of a potentially important placebo effect and the need for further quantitative and qualitative research in CP/CPPS. The observed variability in response to therapy could suggest that CP/CPPS is actually comprised of a number of separate disease entities with discrete causes that require different treatments. Our current understanding of CP/CPPS is not complete enough to allow us to employ appropriate interventions for all patients and it is important to continue to conduct research to improve our understanding of the mechanism and treatment of the disease.

## Supporting Information

Figure S1 **PRISMA 2009 Flow Diagram.**
(TIFF)

Figure S2 **PRISMA 2009 Checklist.**
(PDF)

Table S1 **Search strategy.**
(DOCX)

Table S2 **Baseline Demographic Characteristics of Studies.**
(DOCX)

Table S3 **Jadad Scoring Results.**
(DOCX)

Table S4 **Cochrane Risk of Bias Assessment for randomization, blinding, concealed allocation, intention to treat analysis, other forms of bias, and industry support.**
(DOCX)

Table S5 **Impact of Longer Treatment Duration Among Studies Using Alpha-Blockers.**
(DOCX)

Table S6 **Direct Comparisons of CP/CPPS Treatment Modalities.**
(DOCX)

Table S7 **Direct Comparisons of Alpha-Blocker and Antibiotic Combinations.**
(DOCX)

Table S8 **Sensitivity Analyses.**
(DOCX)

Table S9 **Comparison of Current Classic (Pair-Wise) Meta-analysis and the 2011 Anothaisintawee et al. Network Meta-Analysis.**
(DOCX)
