# Supplementary figures and images for: Therapeutic Intervention for Chronic Prostatitis/Chronic Pelvic Pain Syndrome (CP/CPPS): A Systematic Review and Meta-Analysis
Source: PLoS One. 2012 Aug 1;7(8):e41941. doi: 10.1371/journal.pone.0041941 (PMC3411608; doi:10.1371/journal.pone.0041941)

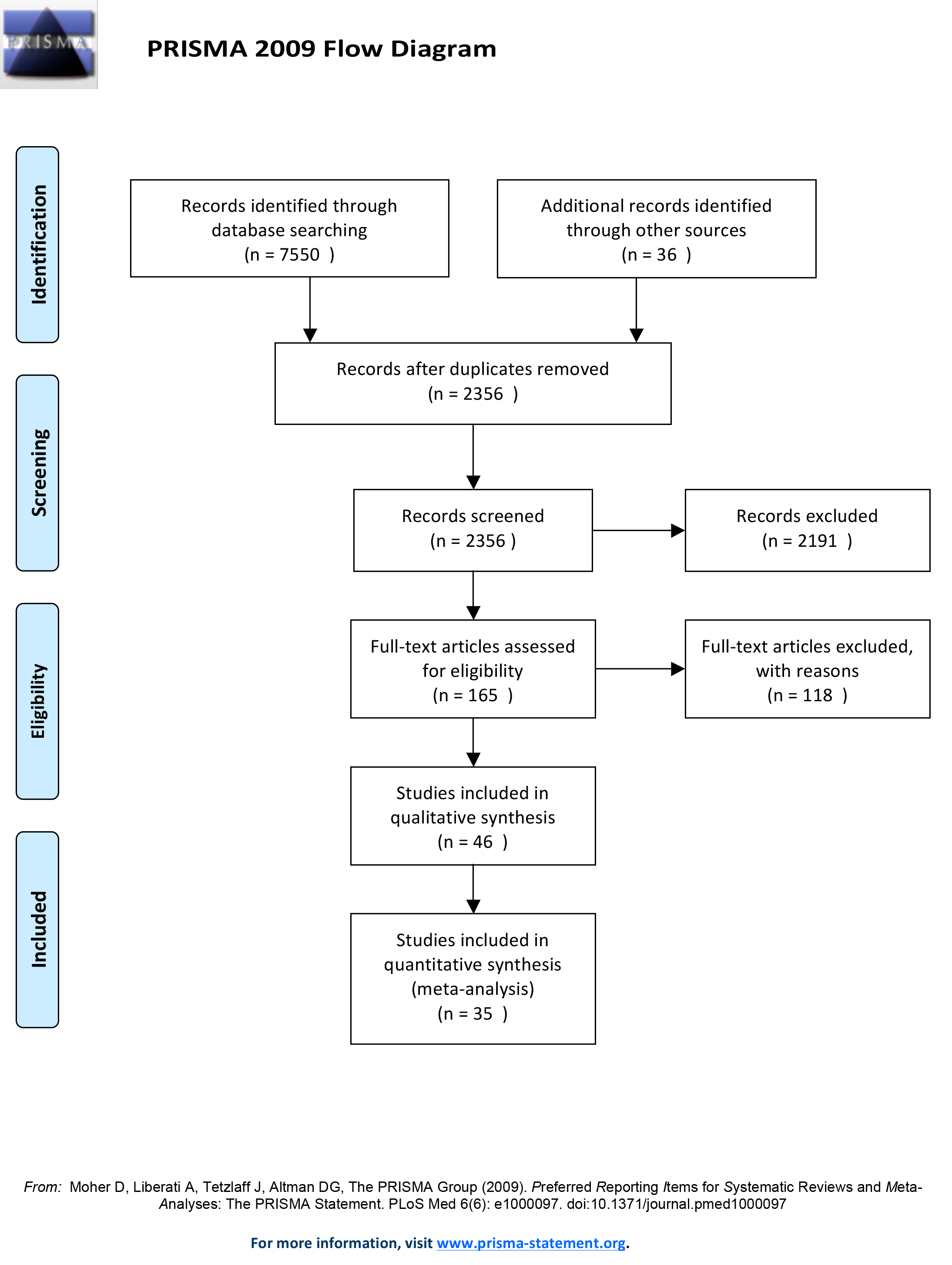

Supplement: Figure S1 — PRISMA 2009 Flow Diagram. (TIFF) [file pone.0041941.s001.tiff]
