# Supplementary material for: Therapeutic Intervention for Chronic Prostatitis/Chronic Pelvic Pain Syndrome (CP/CPPS): A Systematic Review and Meta-Analysis
Source: PLoS One. 2012 Aug 1;7(8):e41941. doi: 10.1371/journal.pone.0041941 (PMC3411608; doi:10.1371/journal.pone.0041941)
Supplement: Table S1 — Search strategy. (DOCX) [file pone.0041941.s003.docx]

**Table S1: PubMed Search Strategy**

#1 "chronic pelvic pain syndrome"[Text Words]

#2 "nonbacterial prostatitis"[Text Words]

#3 "abacterial prostatitis"[Text Words]

#4 "prostatodynia"[Text Words]

#5 "prostatism"[Text Words]

#6 #1 OR #2 OR #3 OR #4 OR #5

#7 "Prostatitis"[Mesh]

#8 "chronic"[Text Words]

#9 #7 AND #8

#10 "Pelvic Pain"[Mesh]

#11 "chronic"[Text Words]

#12 #10 AND #11

#13 #6 OR #9 OR #12

#14 "therapy "[Subheading]

#15 "Anti-Infective Agents"[Pharmacological Action]

#16 "Analgesics"[Mesh]

#17 "Analgesics "[Pharmacological Action]

#18 "Adrenergic alpha-Antagonists"[Pharmacological Action]

#19 "Analgesia"[Mesh]

#20 "Pain/therapy"[Mesh]

#21 "Hyperalgesia"[Mesh]

#22 “allodynia” [Text Words]

#23 #14 OR #15 OR #16 OR #17 OR #18 OR #19 OR #20 OR #21 OR #22

#24 #13 AND #23

Database (Platform: Dates Covered) Types of Sources Indexed

| **Database** | **Platform/Provider** | **Dates Covered** | **Sources Indexed** |
| --- | --- | --- | --- |
| PubMed | NLM | 1947 - 2011 | Journal citations |
| EMBASE | Elsevier | 1973 - 2011 | Journal citations, meeting abstracts |
| CINAHL | EBSCO | 1999 - 4/8/2011 | Journal citations, meeting abstracts, book chapters |
| Cochrane Library | Wiley | 1999 - 2011 | Cochrane database of systematic reviews (CDSR); Database of abstracts of reviews of effectiveness (DARE); and Cochrane review methodology database (CRMD) |
| Web of Science | Institute for Scientific Information | 1900 - 2010 | Journal articles, meeting abstracts |
| Alt.Health Watch Online | EBSCO | 1984 - 2011 | Journal articles, news items |
| PsycInfo | EBSCO | 1800 – 2011 | Journal articles, book chapters |
| Google Scholar | Google | unknown | Biomedical articles, technical papers, patents, grey literature |
| J-East: Bibliography on science and technology in Japan | Japan Science and Technology Agency | 2001 - 2007 | Journal articles |
| Ichushi-Web | Japan Medical Abstracts Society | 1983 - 2011 | Journal articles |
| Ci Nii | National Institute of Informatics Japan | 1948 - 2011 | Journal articles |
| BIOSIS Previews | BIOSIS | 1994 - 2011 | Articles, book chapters, patents, meeting abstracts |
| Dissertations and Theses PQDT) | ProQuest | 1861 - 2011 | Dissertations, theses |
| NLM Gateway Meeting Abstracts | NLM | 1987 - 2011 | Meeting abstracts |
| Conference Papers Index | Cambridge Scientific Abstracts | 1978 - 2011 | Meeting abstracts |
| Clinical Trials.gov | NLM | 2007 - 2011 | Clinical trials |
| Cochrane controlled trials register (CCTR) | Wiley | 1999 - 2011 | Controlled clinical trials |
| Other online trial registries | myRCT, Center Watch, Glaxo Smith Kline, Roche | 1994 - 2011 | Clinical trials |
| National Institute of Diabetes and Digestive and Kidney Diseases (NIDDKD) | http://kidney.niddk.nih.gov/index.aspx |  | Clinical trials, technical papers |
| Factiva | Dow Jones/Reuters | 1969 - 2011 | News, business and financial articles |
