# Supplementary material for: Therapeutic Intervention for Chronic Prostatitis/Chronic Pelvic Pain Syndrome (CP/CPPS): A Systematic Review and Meta-Analysis
Source: PLoS One. 2012 Aug 1;7(8):e41941. doi: 10.1371/journal.pone.0041941 (PMC3411608; doi:10.1371/journal.pone.0041941)
Supplement: Table S2 — Baseline Demographic Characteristics of Studies. (DOCX) [file pone.0041941.s004.docx]

**Table S2. Baseline Demographic Characteristics of Studies**

| **Author** | **Year, Country** | **PubMed ID** | | **Medication/ Intervention** | **Dose** | | | **Duration (wks)** | | **Sample size** | **Age (mean +/- SD)** |
| --- | --- | --- | --- | --- | --- | --- | --- | --- | --- | --- | --- |
| **PLACEBO-CONTROLLED RANDOMIZED CONTROLLED TRIALS** | | | | | | | | | | | |
| **MEDICATIONS** | | | | | | | | | | | |
| **Alpha-Blockers** | | | | | | | | | | | |
| Alexander RB  *et al.*,[18] | 2004, USA and Canada | 15492337 | Ciprofloxacin vs. Tamsulosin | | | 500 mg BID vs. 0.4 mg QD | 6 | | 49 | | 44.6 (N/A) |
|  | | | Ciprofloxacin vs. Ciprofloxacin + Tamsulosin | | | 500 mg BID vs. 500 mg BID + 0.4 mg QD | 6 | | 49 | | 44.6 (N/A) |
|  | | | Tamsulosin vs. Ciprofloxacin + Tamsulosin | | | 0.4 mg QD vs. 500 mg BID + 0.4 mg QD | 6 | | 49 | | 44.6 (N/A) |
| Cheah PY *et al.*, [19] | 2003, Malaysia | 12544314 | Terazosin vs. | | | Dose escalation from 1 to 5 mg QD | 14 | | 43 | | 35.5 (range, 20-50) |
|  | | | Placebo | | |  | 14 | | 43 | | 35.5 (range, 20-50) |
| Mehik *et al.*, [20] | 2003, Finland | 12946740 | Alfuzosin vs. | | | 5 mg BID | 24 | | 19 | | 49.5 |
|  | | | Placebo | | |  | 24 | | 21 | | 49.5 |
| Nickel *et al.*, [21] | 2004, USA and Canada | 15017228 | Tamsulosin vs. | | | 0.4 mg QD | 6 | | 27 | | 40.8 (21-56) |
|  | | | Placebo | | |  | 6 | | 30 | | 40.8 (21-56) |
| Nickel *et al.,* [22] | 2008, USA and Canada | 19092152 | Alfuzosin vs. | | | 10 mg QD | 12 | | 138 | | 40.1 (1.4) |
|  | | | Placebo | | |  | 12 | | 134 | | 40.1 (1.4) |
| Nickel *et al.*, [23] | 2011, USA and Canada | 21571345 | Silodosin vs. | | | 4 mg QD | 12 | | 52 | | 48.2 (N/A) |
|  | | | Silodosin vs. | | | 8 mg QD | 12 | | 45 | | 48.2 (N/A) |
|  | | | Placebo | | |  | 12 | | 54 | | 48.2 (N/A) |
| Sivkov *et al.*, [24] | 2005, Russia | 15776832 | Terazosin vs. | | | 5 mg QD | 8 | | 29 | | N/A |
|  | | | Placebo | | |  | 8 | | 22 | | N/A |
| Tugcu *et al.,* [25] | 2007, Turkey | 17084960 | Doxazosin vs. | | | 4 mg QD | 24 | | 30 | | 29.1 (5.2) |
|  | | | Placebo | | |  | 24 | | 30 | | 29.1 (5.2) |
| **Antibiotics** | | | | | | | | | | | |
| Nickel *et al.,* [26] | 2003, USA and Canada | 14550427 | Levofloxacin vs. | | | 500 mg QD | 6 | | 45 | | 56.1 (range, 36-78) |
|  | | | Placebo | | |  | 6 | | 35 | | 56.1 (range, 36-78) |
| **Finasteride** | | | | | | | | | | | |
| Nickel *et al.,* [27] | 2004, USA and Canada | 15142149 | Finasteride vs. | | | 5 mg QD | 24 | | 33 | | 44.4 (0.5) |
|  | | | Placebo | | |  | 24 | | 31 | | 44.4 (0.5) |
| **Glycosaminoglycan** | | | | | | | | | | | |
| Nickel *et al.,* [28] | 2005, USA and Canada | 15758763 | Pentosan polysulfate vs. | | | 300 mg TID | 16 | | 51 | | 39.2 (range, 21-59) |
|  | | | Placebo | | |  | 16 | | 49 | | 39.2 (range, 21-59) |
| **Mepartricin** | | | | | | | | | | | |
| De Rose *et al.*, [29] | 2004, Italy | 14751338 | Mepartricin vs. | | | 40 mg QD | 60 days | | 13 | | 33 (range, 26-52) |
|  | | | Placebo | | |  | 60 days | | 13 | | 33 (range, 26-52) |
| **NSAIDs** | | | | | | | | | | | |
| Nickel *et al.,* [30] | 2003, USA and Canada | 12629372 | Rofecoxib vs. | | | 25 or 50 mg QD | 6 | | 102 | | 45.9 (range, 19-80) |
|  | | | Placebo | | |  | 6 | | 59 | | 45.9 (range, 19-80) |
| Zhao *et al.,* [31] | 2009, China | 19787151 | Celecoxib vs. | | | 200 mg QD | 6 | | 32 | | N/A |
|  | | | Placebo | | |  | 6 | | 32 | | N/A |
| **Pollen extracts** | | | | | | | | | | | |
| Wagenlehner *et al.,* [32] | 2009, Germany | 19524353 | *Secale cereale* (Cernitin) pollen extract vs. | | | 2 capsules (63 mg of the defined pollen extract fractions Cernitin T60) q 8 hrs | 12 | | 70 | | 39.5 (8.1) |
|  | | | Placebo | | |  | 12 | | 69 | | 39.5 (8.1) |
| **Pregabalin** | | | | | | | | | | | |
| Pontari *et al.,* [33] | 2010, USA and Canada | 20876412 | Pregabalin vs. | | | Titrated 150-600 mg QD for 2 weeks | 6 | | 217 | | 47 (13.1) |
|  | | | Placebo | | |  | 6 | | 104 | | 47 (13.1) |
| **INTERVENTIONS** | | | | | | | | | | | |
| **Acupuncture** | | | | | | | | | | | |
| Lee, SWH *et al.,* [34] | 2008,  Malaysia, USA | 18187077 | Acupuncture | | | 30 minutes twice weekly | 10 | | 44 | | 40.9 (11.0)* |
|  | | | Sham Acupuncture | | | 30 minutes twice weekly | 10 | | 45 | | 42.8 (9.4)* |
| **Aerobic Exercise** | | | | | | | | | | | |
| Giubilei *et al.*, [35] | 2007, Italy | 17162029 | Aerobic Exercise | | | 3 times a week | 18 | | 48 | | 37.58 (7.80) |
|  | | | Non-Aerobic Stretching and Motion Exercises | | | 3 times a week | 18 | | 49 | | 35.88 (8.45) |
| **Extracorporeal Shock Wave Therapy (ESWT)** | | | | | | | | | | | |
| Zimmermann *et al.,* [36] | 2009, Austria, Romania | 19372000 | ESWL | | | 3000 pulses at 3 Hz | 4 | | 30 | | 42.5 (N/A) |
|  | | | Sham ESWL | | | 3000 sham pulses | 4 | | 30 | | 42.5 (N/A) |
| **Posterior tibial nerve stimulation (PTNS)** | | | | | | | | | | | |
| Kabay *et al.*, [37] | 2009, Turkey | 19641356 | PTNS | | | Maximum tolerable | 12 | | 45 | | 37.7 (7.4) |
|  | | | Sham | | | Maximum tolerable sham | 12 | | 44 | | 37.7 (7.4) |

| **NON PLACEBO-CONTROLLED RANDOMIZED CONTROLLED TRIALS** | | | | | | | |
| --- | --- | --- | --- | --- | --- | --- | --- |
| **Author** | **Year, Country** | **PubMed ID** | **Medication/ Intervention** | **Dose** | **Duration (wks)** | **Sample size** | **Age (mean +/- SD)** |
| Cha WH *et al.*, [38] | 2009 | No PMID | Levofloxacin | 300 mg TID | 8 | 36 | 39.28 (N/A) |
|  | |  | Levofloxacin +  Alfuzosin | 300 mg TID + 10 mg QD | 8 | 33 | 39.28 (N/A) |
|  | |  | Levofloxacin + Rowatinex Terpene Mixture | 300 mg TID + three capsules TID | 8 | 34 | 39.28 (N/A) |
| Jeong CW *et al.*, [39] | 2008  Korea | 18362485 | Levofloxacin + Doxazosin | 100 mg BID + 4 mg QD | 6 | 29 | 40.1 (range, 23-60) |
|  | |  | Levofloxacin | 100 mg BID | 6 | 26 | 40.1 (range, 23-60) |
|  | |  | Doxazosin | 4 mg QD | 6 | 26 | 40.1 (range, 23-60) |
| Jung YH *et al.*, [40] | 2006,  South Korea | No PubMed ID | Levofloxacin + Talmiflunate | 300 mg QD+  three capsules QD | 12 | 56 | 33.6 (N/A) |
|  | |  | Levofloxacin + Talmiflunate + Terazosin | 300 mg QD+ three capsules QD + 3.5 mg QD | 12 | 71 | 33.6 (N/A) |
| Kaplan SA *et al.*, [41] | 2004, USA | 14665895 | Saw palmetto | 325 mg QD | 52 | 32 | 43.2 (N/A) |
|  | |  | Finasteride | 5 mg QD | 52 | 32 | 43.2 (N/A) |
| Lee CB *et al.*, [42] | 2006, South Korea | 16418872 | Rowatinex | 200 mg TID | 6 | 25 | 43.2 (N/A) |
|  | |  | Ibuprofen | 600 mg TID | 6 | 25 | 43.2 (N/A) |
| Li B *et al.*, [43] | 2007, China | 17432691 | Chinese herbal remedies (Tiaoshen Tonglin Decoction) | BID | 60 days | 56 | 29.97 (N/A) |
|  | |  | Terazosin | 2 mg QD | 60 days | 52 | 29.97 (N/A) |
| Morgia G *et al.*, [44] | 2010, Italy | 20332612 | Profluss (serenoa repens + lycopene + seleniated sodium) | 320 mg QD + 5 mg QD + 50 μg QD | 8 | 51 | 38.43 (7.5) |
|  | | | Serenoa repens | 320 mg QD | 8 | 51  51 | 38.43 (7.5) |
| Paick JS *et al.*, [45] | 2006, South Korea | 16683008 | Terazosin | 2-4 mg QD | 6 | 21 | 45.7 (N/A) |
|  | | | Terazosin+  Extracorporeal magnetic innervation (ExMI) | 2-4 mg QD + 10 Hz for 10 mins & 50 Hz for 10 mins | 6 | 19 | 45.7 (N/A) |
| Shen SL *et al.*, [46] | 2006  China | 17009541 | Chinese herbal remedies + weekly prostatic massage | BID +  once a week | 8 | 40 | 31 (N/A) |
|  | | | Chinese herbal remedies | BID | 8 | 32 | 31 (N/A) |
| Tan Y *et al.*, [47] | 2009, China | No PubMed ID | Tamsulosin+  Prostant | 0.2 mg QD +  2 grams QD | 6 | 45 | 33.28 (N/A) |
|  | | | Terazosin | 2 mg QD | 6 | 45 | 33.28 (N/A) |
| Ye ZQ *et al.*, [48] | 2008, China | 18380933 | Tamsulosin + levofloxacin | 0.2 mg QD + 0.2 grams QD | 12 | 42 | N/A |
|  | | | Tamsulosin | 0.2 mg QD | 12 | 21 | N/A |
|  | | | Levofloxacin | 0.2 grams QD | 12 | 21 | N/A |
| Youn CW *et al.,* [49] | 2008, South Korea | No PMID | Gatifloxacin | 200 mg BID | 6 | 35 | 41.6 |
|  | | | Gatifloxacin + Doxazosin | 200 mg BID + 1 pill QD | 6 | 34 | 41.6 |
| Ziaee *et al.,* [50] | 2006, Iran | 16650295 | Allopurinol + ofloxacin | 100 mg q 8hrs + 200 mg TID | 12 | 29 | 33.39 (N/A) |
|  | | | Ofloxacin | 200 mg TID | 12 | 27 | 33.39 (N/A) |
| Zeng X *et al.*, [51] | 2004, China | 15148925 | Celecoxib | 200 mg QD | 6 | 32 | 35.6 (N/A) |
|  | | | Celecoxib | 200 mg BID | 6 | 32 | 35.6 (N/A) |
| Zhou *et al.,* [52] | 2008, China | 18538692 | Tetracycline HCl + vitamin C + co-vitamin B | 500 mg QD + 0.4 g QD + 0.2 g QD | 12 | 24 | N/A |
|  | | | Placebo: co-vitamin B | 0.2 g QD | 12 | 24 | N/A |
