# Supplementary material for: Therapeutic Intervention for Chronic Prostatitis/Chronic Pelvic Pain Syndrome (CP/CPPS): A Systematic Review and Meta-Analysis
Source: PLoS One. 2012 Aug 1;7(8):e41941. doi: 10.1371/journal.pone.0041941 (PMC3411608; doi:10.1371/journal.pone.0041941)
Supplement: Table S3 — Jadad Scoring Results. (DOCX) [file pone.0041941.s005.docx]

**Table S3: Jadad Scoring Results**

| Author, Year | | PMID | Jadad Total Score | Randomized | Randomization Appropriate | Blinded | Blinding Appropriate | Withdrawals Reported | Statistics Reported | Inclusion and Exclusion Criteria | Adverse Events Reported |
| --- | --- | --- | --- | --- | --- | --- | --- | --- | --- | --- | --- |
| **Alpha-blockers** | | | | | | | | | | | |
| Alexander RB, 2004 [18] | | 15492337 | 8 | 1 | 1 | 1 | 1 | 1 | 1 | 1 | 1 |
| Cheah PY, 2003 [19] | | 12544314 | 6 | 1 | -1 | 1 | 1 | 1 | 1 | 1 | 1 |
| Mehik A, 2003 [20] | | 12946740 | 5 | 1 | -1 | 1 | 1 | 1 | 1 | 1 | 0 |
| Nickel JC, 2004 [21] | | 15142149 | 5 | 1 | -1 | 1 | 1 | 0 | 1 | 1 | 1 |
| Nickel JC, 2008 [22] | | 19092152 | 8 | 1 | 1 | 1 | 1 | 1 | 1 | 1 | 1 |
| Nickel JC, 2011 [23] | | 21571345 | 8 | 1 | 1 | 1 | 1 | 1 | 1 | 1 | 1 |
| Sivkov et al, 2005 [24] | | 15776832 | 8 | 1 | 1 | 1 | 1 | 1 | 1 | 1 | 1 |
| Tugcu V, 2007 [25] | | 17084960 | 4 | 1 | -1 | 0 | n/a | 1 | 1 | 1 | 1 |
| **Antibiotics** | | | | | | | | | | | |
| Nickel JC, 2003 [26] | | 12629372 | 8 | 1 | 1 | 1 | 1 | 1 | 1 | 1 | 1 |
| **Finasteride** | | | | | | | | | | | |
| Nickel JC, 2004 [27] | | 15017228 | 4 | 1 | -1 | 1 | -1 | 1 | 1 | 1 | 1 |
| **Glycosaminoglycan** | | | | | | | | | | | |
| Nickel JC, 2005 [28] | | 15758763 | 6 | 1 | -1 | 1 | 1 | 1 | 1 | 1 | 1 |
| **Mepartricin** | | | | | | | | | | | |
| De Rose AF, 2004 [29] | | 14751338 | 6 | 1 | 1 | 1 | -1 | 1 | 1 | 1 | 1 |
| **NSAIDs** | | | | | | | | | | | |
| Nickel JC, 2003 [30] | | 14550427 | 8 | 1 | 1 | 1 | 1 | 1 | 1 | 1 | 1 |
| Zhao WP, 2009 [31] | | 19787151 | 8 | 1 | 1 | 1 | 1 | 1 | 1 | 1 | 1 |
| **Pollen extracts** | | | | | | | | | | | |
| Wagenlehner FME,  2009 [32] | | 19524353 | 8 | 1 | 1 | 1 | 1 | 1 | 1 | 1 | 1 |
| **Pregabalin** | | | | | | | | | | | |
| Pontari MA, 2010 [33] | | 20876412 | 8 | 1 | 1 | 1 | 1 | 1 | 1 | 1 | 1 |
| **Interventions** | | | | | | | | | | | |
| **Acupuncture** | | | | | | | | | | | |
| Lee SWH, 2008 [34] | | 18187077 | 7 | 1 | 1 | 1 | 1 | 0 | 1 | 1 | 1 |
| **Aerobic Exercise** | | | | | | | | | | | |
| Giubilei G, 2007 [35] | | 17162029 | 6 | 1 | -1 | 1 | 1 | 1 | 1 | 1 | 1 |
| **Extracorporeal Shock Wave Therapy (ESWT)** | | | | | | | | | | | |
| Zimmermann R, 2009 [36] | | 19372000 | 6 | 1 | -1 | 1 | 1 | 1 | 1 | 1 | 1 |
| **Posterior tibial nerve stimulation (PTNS)** | | | | | | | | | | | |
| Kabay S, 2009 [37] | | 19641356 | 4 | 1 | -1 | 1 | 1 | 0 | 1 | 1 | 0 |
| **Non-placebo-controlled trials** | | | | | | | | | | | |
| Cha WH, 2009 [38] | | No PMID | 4 | 1 | -1 | 0 | n/a | 1 | 1 | 1 | 1 |
| Jeong CW, 2008 [39] | | 18362485 | 4 | 1 | -1 | 0 | n/a | 1 | 1 | 1 | 1 |
| Jung YH, 2006 [40] | | No PMID | 3 | 1 | -1 | 0 | n/a | 1 | 1 | 1 | 0 |
| Kaplan SA, 2004 [41] | | 14665895 | 3 | 1 | -1 | 0 | n/a | 0 | 1 | 1 | 1 |
| Lee CB, 2006 [42] | | 16418872 | 4 | 1 | -1 | 0 | n/a | 1 | 1 | 1 | 1 |
| Li B, 2007 [43] | | 17432691 | 4 | 1 | 1 | 0 | n/a | 0 | 1 | 1 | 0 |
| Morgia G, 2010 [44] | | 20332612 | 8 | 1 | 1 | 1 | 1 | 1 | 1 | 1 | 1 |
| Paick JS, 2006 [45] | | 16683008 | 5 | 1 | 1 | 0 | n/a | 1 | 1 | 1 | 0 |
| Shen SL, 2006 [46] | | 17009541 | 2 | 1 | -1 | 0 | n/a | 0 | 1 | 1 | 0 |
| Tan Y, 2009 [47] | | No PMID | 4 | 1 | -1 | 0 | n/a | 1 | 1 | 1 | 1 |
| Ye ZQ, 2008 [48] | | 18380933 | 3 | 1 | -1 | 0 | n/a | 1 | 1 | 1 | 0 |
| Youn CW, 2008 [49] | | No PMID | 3 | 1 | -1 | 0 | n/a | 1 | 1 | 1 | 0 |
| Ziaee AM, 2006 [50] | | 16650295 | 6 | 1 | -1 | 1 | 1 | 1 | 1 | 1 | 1 |
| Zeng X, 2004 [51] | | 15148925 | 3 | 1 | -1 | 0 | n/a | 1 | 1 | 1 | 0 |
| Zhou Z, 2008 [52] | | 18538692 | 2 | 1 | -1 | 0 | n/a | 0 | 1 | 1 | 0 |
|  |  |  |  |  |  |  |  |  |  |  |  |
|  |  | Mean | 5.4 |  |  |  |  |  |  |  |  |
|  |  | Median | 5 |  |  |  |  |  |  |  |  |
|  |  | Range | 2 to 8 |  |  |  |  |  |  |  |  |
|  |  | 6 or Greater | 15 |  |  |  |  |  |  |  |  |
|  |  | 4 or lower | 15 |  |  |  |  |  |  |  |  |

The highest Jadad Score is 8 based on randomization, blinding, withdrawal reporting, statistics reporting, clearly defined inclusion and exclusion criteria, and adverse event reporting.[9] \\
