# Supplementary material for: Therapeutic Intervention for Chronic Prostatitis/Chronic Pelvic Pain Syndrome (CP/CPPS): A Systematic Review and Meta-Analysis
Source: PLoS One. 2012 Aug 1;7(8):e41941. doi: 10.1371/journal.pone.0041941 (PMC3411608; doi:10.1371/journal.pone.0041941)
Supplement: Table S4 — Cochrane Risk of Bias Assessment for randomization, blinding, concealed allocation, intention to treat analysis, other forms of bias, and industry support. (DOCX) [file pone.0041941.s006.docx]

**Table S4. Cochrane Risk of Bias Assessment for randomization, blinding, concealed allocation, intention to treat analysis, other forms of bias, and industry support.**

| **Jadad Issues/Intention-to-treat Study Information** | | | | | | | | | | **Cochrane Risk of Bias Criteria** | | | | | | |
| --- | --- | --- | --- | --- | --- | --- | --- | --- | --- | --- | --- | --- | --- | --- | --- | --- |
| **Author Year** | | **PMID** | | | **Jadad Total Score** | | | **Jadad Quality Problems** | **Intention to Treat** | **Adequate sequence generation** | **Adequate concealed allocation** | **Adequate Blinding** | **Incomplete outcome data addressed** | **Free of selective outcome reporting** | **Free of “other” bias** | **Industry Sponsored** |
| **Alpha-blockers** | | | | | | | | | | | | | | | | |
| Alexander RB,  2004 [18] | | 15492337 | | | 8 | | | None | Yes | Yes | Yes | Yes | Yes | Yes | Yes | No |
| Cheah PY, 2003 [19] | | 12544314 | | | 6 | | | Inappropriate Randomization | No | Yes | Unclear | Yes | Yes | Yes | Yes | Yes |
| Mehik A,  2003 [20] | | 12946740 | | | 5 | | | Inappropriate Randomization | No | Unclear | Unclear | Yes | Yes | Yes | No | Yes |
| Nickel JC,  2004 [21] | | 15142149 | | | 5 | | | Inappropriate randomization Inadequate withdrawal reporting | No | Unclear | Unclear | Yes | Yes | Yes | Yes | Yes |
| Nickel JC,  2008 [22] | | 19092152 | | | 8 | | | None | Yes | Yes | Yes | Yes | Yes | Yes | Yes | No |
| Nickel JC,  2011 [23] | | 21571345 | | | 8 | | | None | Yes | Yes | Yes | Yes | Yes | Yes | Yes | Unclear |
| Sivkov et al, 2005 [24] | | 15776832 | | | 8 | | | None | No | Yes | Yes | Yes | No | Yes | Yes | Unclear |
| Tugcu V,  2007 [25] | | 17084960 | | | 4 | | | Inappropriate randomization Inadequate blinding | Yes | Unclear | Unclear | Yes | Yes | Yes | Yes | Unclear |
| **Antibiotics** | | | | | | | | | | | | | | | | |
| Nickel JC,  2003 [26] | | 12629372 | | | 8 | | | None | Yes | Yes | Yes | Yes | Yes | Yes | Yes | Yes |
| **Finasteride** | | | | | | | | | | | | | | | | |
| Nickel JC,  2004 [27] | | 15017228 | | | 4 | | | Inappropriate randomization  Inadequate blinding | Yes | Yes | Unclear | Yes | Yes | Yes | Yes | Yes |
| **Glycosaminoglycan** | | | | | | | | | | | | | | | | |
| Nickel JC,  2005 [28] | | 15758763 | | | 6 | | Inappropriate randomization | | Yes | Yes | Unclear | Yes | No | Yes | Yes | Yes |
| **Mepartricin** | | | | | | | | | | | | | | | | |
| De Rose AF,  2004 [29] | | 14751338 | | | 6 | | Inadequate Blinding | | Yes | Yes | Unclear | No | Yes | Yes | Yes | No |
| **NSAIDs** | | | | | | | | | | | | | | | | |
| Nickel JC,  2003 [30] | | 14550427 | | 8 | | | None | | Yes | Yes | Unclear | Yes | Yes | Yes | Yes | Yes |
| Zhao WP,  2009 [31] | | 19787151 | | 8 | | | None | | Yes | Yes | Unclear | Yes | Yes | Yes | Yes | Unclear |
| **Pollen extracts** | | | | | | | | | | | | | | | | |
| Wagenlehner FME,  2009 [32] | | 19524353 | | 4 | | | None | | Yes | Yes | Unclear | Yes | No | Yes | Yes | Yes |
| **Pregabalin** | | | | | | | | | | | | | | | | |
| Pontari MA,  2010 [33] | | 20876412 | | 8 | | | None | | Yes | Yes | Yes | Yes | Yes | No | Yes | No |
| **Interventions** | | | | | | | | | | | | | | | | |
| **Acupuncture** | | | | | | | | | | | | | | | | |
| Lee SWH,  2008 [34] | | 18187077 | | 7 | | | Inadequate withdrawal reporting | | Yes | Yes | Unclear | Yes | No | Yes | Yes | Unclear |
| **Aerobic Exercise** | | | | | | | | | | | | | | | | |
| Giubilei G,  2008 [35] | | 17162029 | | 6 | | | Inappropriate randomization | | Yes | No | Unclear | Yes | Yes | Yes | Yes | Unclear |
| **Extracorporeal Shock Wave Therapy (ESWT)** | | | | | | | | | | | | | | | | |
| Zimmermann R,  2009 [36] | | 19372000 | | 8 | | | Inappropriate randomization | | Yes | No | Unclear | Yes | Yes | Yes | Yes | No |
| **Posterior tibial nerve stimulation (PTNS)** | | | | | | | | | | | | | | | | |
| Kabay S,  2009 [37] | 19641356 | | 4 | | | Inappropriate randomization  Inadequate withdrawal reporting  Inadequate reporting of adverse events | | | Yes | No | Unclear | No | No | Yes | Yes | Unclear |
| **Non-placebo controlled trials** | | | | | | | | | | | | | | | | |
| Cha WH, 2009 [38] | No PMID | | 4 | | | Inappropriate randomization  Inadequate blinding  Inadequate reporting of adverse events | | | Yes | No | Unclear | N/A | Yes | Yes | Yes | Unclear |
| Jeong CW,  2008 [39] | 18362485 | | 4 | | | Inappropriate randomization  Inadequate blinding | | | Yes | Unclear | Unclear | Unclear | Yes | Yes | Yes | Unclear |
| Jung YH,  2006 [40] | No PMID | | 3 | | | Inappropriate randomization  Inadequate blinding  Inadequate reporting of adverse events | | | Yes | No | Unclear | N/A | Yes | Yes | Yes | Unclear |
| Kaplan SA,  2004 [41] | 14665895 | | 3 | | | Inappropriate randomization  Inadequate blinding  Inadequate withdrawal reporting | | | Yes | No | Unclear | N/A | No | Yes | Yes | Unclear |
| Lee CB,  2006 [42] | 16418872 | | 4 | | | Inappropriate randomization  Inadequate blinding | | | Yes | No | Unclear | N/A | Yes | Yes | Yes | Unclear |
| Li B,  2007 [43] | 17432691 | | 4 | | | Inadequate blinding  Inadequate withdrawal reporting  Inadequate reporting of adverse events | | | No | Yes | Unclear | No | Yes | Yes | Yes | Unclear |
| Morgia G,  2010 [44] | 20332612 | | 6 | | | None | | | Yes | Yes | Unclear | Yes | Yes | Yes | Yes | Unclear |
| Paick JS,  2006 [45] | 16683008 | | 5 | | | Inadequate blinding  Inadequate reporting of adverse events | | | Yes | Yes | Unclear | N/A | Yes | Yes | Yes | Unclear |
| Shen SL,  2006 [46] | 17009541 | | 2 | | | Inappropriate randomization  Inadequate blinding  Inadequate withdrawal reporting  Inadequate reporting of adverse events | | | No | No | Unclear | N/A | Yes | Yes | Yes | Unclear |
| Tan Y,  2009 [47] | No PMID | | 4 | | | Inappropriate randomization  Inadequate blinding | | | No | No | Unclear | N/A | Yes | Yes | Yes | Unclear |
| Ye ZQ,  2008 [48] | 18380933 | | 3 | | | Inappropriate randomization  Inadequate blinding  Inadequate reporting of adverse events | | | Yes | Unclear | Unclear | No | Unclear | Yes | No | No |
| Youn CW,  2008 [49] | No PMID | | 2 | | | Inappropriate randomization  Inadequate blinding  Inadequate reporting of adverse events | | | Yes | No | Unclear | N/A | Yes | Yes | Yes | Unclear |
| Ziaee AM,  2006 [50] | 16650295 | | 6 | | | Inappropriate randomization | | | Yes | No | Unclear | Yes | Yes | Yes | Yes | Unclear |
| Zeng X,  2004 [51] | 15148925 | | 3 | | | Inappropriate randomization  Inadequate blinding  Inadequate withdrawal reporting | | | No | No | Unclear | N/A | Yes | Yes | Yes | Unclear |
| Zhou Z,  2008 [52] | 18538692 | | 3 | | | Inappropriate randomization  Inadequate blinding  Inadequate withdrawal reporting  Inadequate reporting of adverse events | | |  | Unclear | Unclear | Unclear | Unclear | Yes | No | Unclear |
