# Supplementary material for: Therapeutic Intervention for Chronic Prostatitis/Chronic Pelvic Pain Syndrome (CP/CPPS): A Systematic Review and Meta-Analysis
Source: PLoS One. 2012 Aug 1;7(8):e41941. doi: 10.1371/journal.pone.0041941 (PMC3411608; doi:10.1371/journal.pone.0041941)
Supplement: Table S5 — Impact of Longer Treatment Duration Among Studies Using Alpha-Blockers. (DOCX) [file pone.0041941.s007.docx]

**Table S5. Impact of Longer Treatment Duration Among Studies Using Alpha-Blockers**

| **Domain** | **Beta-Coefficient (95% CI)** |
| --- | --- |
| NIH-CPSI Total Score | 0.19 (0.11-0.27) |
| Pain | 0.07 (0.03-0.11) |
| Voiding | 0.08 (0.04-0.12) |
| Quality of Life | 0.07 (0.03-0.11) |
