# Supplementary material for: Therapeutic Intervention for Chronic Prostatitis/Chronic Pelvic Pain Syndrome (CP/CPPS): A Systematic Review and Meta-Analysis
Source: PLoS One. 2012 Aug 1;7(8):e41941. doi: 10.1371/journal.pone.0041941 (PMC3411608; doi:10.1371/journal.pone.0041941)
Supplement: Table S6 — Direct Comparisons of CP/CPPS Treatment Modalities. (DOCX) [file pone.0041941.s008.docx]

**Table S6. Direct Comparisons of CP/CPPS Treatment Modalities**

| **Author, year** | **Comparison** | **NIH-CPSI Total Score** | **Pain Domain Subscore** | **Voiding Domain Subscore** | **QOL Domain Subscore** |
| --- | --- | --- | --- | --- | --- |
| Zeng X, 2004 [51] | **200 mg vs. 400 mg Celecoxib** | **5.2 (4.5-5.9)** | **3.0 (2.5-3.5)** | **2.1 (1.59-2.60)** | 0.10 (-0.81 to 1.01) |
| Tugcu V, 2007 [25] | **Doxazosin vs. Doxazosin + Ibuprofen + Thiocolchicoside** | 0.80 (-2.42 to 4.02) | 0.000 ( -2.4 to 2.4) | -1 0 (-3.22 to 1.02) | 1.90 (-0.77 to 4.57) |
| Tan Y, 2009 [47] | **Terazosin vs. Tamsulosin + Prostant** | **5.96 (3.14-8.78)** | **1.51 (0.12-2.89)** | **2.54 (1.34-3.74)** | **1.91 (0.82 – 2.99)** |
| Shen SL, 2006 [46] | **Chinese Herbal Remedies vs. Chinese Herbal Remedies + Massage** |  |  | **2.0 (1.21-2.79)** | **2.9 (2.18-3.62)** |
| Nickel JC, 2011 [23] | **4 mg vs. 8 mg Silodosin** | -2.70 (-6.9 to 1.46) | -1.10 (-3.32 to 1.12) | -0.80 (-1.97 to 0.37) | -1.0 (-2.41 to 0.41) |
| Paick JS, 2006 [45] | **Terazosin vs. Terazosin + EMI** | 3.0 (-1.84 to 7.84) | 2.50 (-1.84 to 6.84) | -0.50 (-2.08 to 1.08) | -0.5 (-2.47 to 1.47) |
| Li B, 2007 [43] | **Terazosin vs. Chinese Herbal Remedies** | **3.8 (2.78-4.82)** | **2.6 (1.03-4.17)** | **1.1 (0.36-1.84)** | 0.50 (-0.80 to 1.80) |
| Cha WH, 2009 [38] | **Levofloxacin vs. Levofloxacin + Rowatinex Terpene Mixture** | 0.41 (-0.06 to 0.88) | 0.28 (-0.19 to 0.75) | 0.47 (-0.01 to 0.94) | 0.23 (-0.24 to 0.70) |
| Cha WH, 2009 [38] | **Levofloxacin + Alfuzosin vs. Levofloxacin + Rowatinex Terpene Mixture** | 0.08 (-0.40 to 0.55) | 0.34 (-0.14 to 0.82) | -0.59 (-1.08 to -0.10) | -0.09(-0.57 to0.39) |
| Lee CB, 2006 [42] | **Ibuprofen vs. Rowatinex Terpene Mixture** | 2.2 (-0.02 to 4.42) | 1.10 (-0.23 to 2.43) | 0.0 (-0.45 to 0.45) | 0.30 (-0.53 to 1.1) |
| Kaplan SA, 2004 [41] | **Saw Palmetto vs. Finasteride** | **6.5 (3.62 to 9.38)** | 0.80 (-0.85 to 2.45) | 0.10 (-1.03 to 1.23) | **2.3 (1.2 to 3.4)** |
| Morgia G, 2010 [44] | **Saw Palmetto vs. Saw Palmetto + Lycopene + Selenium** |  |  |  | **4.98 (2.41-7.55)** |
| Jung YH, 2006 [40] | **Levofloxacin + Tamsulosin vs. Levofloxacin + Tamsulosin + Terazosin** | **5.3 (3.11-7.49)** | **2.90 (1.37-4.43)** | **1.1 (0.38–1 .82)** | **1.3 (0.28 – 2.32)** |
