# Supplementary material for: Therapeutic Intervention for Chronic Prostatitis/Chronic Pelvic Pain Syndrome (CP/CPPS): A Systematic Review and Meta-Analysis
Source: PLoS One. 2012 Aug 1;7(8):e41941. doi: 10.1371/journal.pone.0041941 (PMC3411608; doi:10.1371/journal.pone.0041941)
Supplement: Table S7 — Direct Comparisons of Alpha-Blocker and Antibiotic Combinations. (DOCX) [file pone.0041941.s009.docx]

**Table S7. Direct Comparisons of Alpha-Blocker and Antibiotic Combinations**

| **Comparison** | **NIH-CPSI Total Score** | **Pain Domain Subscore** | **Voiding Domain Subscore** | **QOL Domain Subscore** |
| --- | --- | --- | --- | --- |
| **Antibiotics vs. Alpha-Blockers** [18, 39, 48] | -1.89 (-7.5 to 3.67) (n=3 studies) | 0.96 (-1.31 to 3.23) (n=2 studies) | -0.04 (-1.41 to 1.34) (n=2 studies) | 0.22 (-2.40 to 2.84) (n=2 studies) |
| **Antibiotics vs. Antibiotics + Alpha-Blockers** [18, 39, 40, 48, 49] | 1.38 (0.54 to 3.30) (n=5 studies) | 0.74 (-0.85 to 2.33) (n=4 studies) | 0.54 (0.00 to 1.08) (n=4 studies) | 0.64 (-0.26 to 1.54) (n=4 studies) |
| **Alpha-Blockers vs. Antibiotics + Alpha-Blockers** [18, 39, 48] | 1.78 (-0.85 to 4.41) (n=3 studies) | 0.23 (-2.43 to 2.9) (n=2 studies) | 0.27 (-0.30 to 0.85) (n=2 studies) | 0.51 (-0.52 to 1.54) (n=2 studies) |
