# Supplementary material for: Therapeutic Intervention for Chronic Prostatitis/Chronic Pelvic Pain Syndrome (CP/CPPS): A Systematic Review and Meta-Analysis
Source: PLoS One. 2012 Aug 1;7(8):e41941. doi: 10.1371/journal.pone.0041941 (PMC3411608; doi:10.1371/journal.pone.0041941)
Supplement: Table S8 — Sensitivity Analyses. (DOCX) [file pone.0041941.s010.docx]

**Table S8. Sensitivity Analyses**

| Criteria | NIH-CPSI Total Score | NIH-CPSI Pain Score | NIH-CPSI Voiding Score | NIH-CPSI Quality of Life Score |
| --- | --- | --- | --- | --- |
|  | Coefficient (95% CI), p | Coefficient (95% CI), p | Coefficient (95% CI), p | Coefficient (95% CI), p |
| All Studies | | | | |
| Publication bias (overall) | -1.1 (-3.4 to 1.3), p=0.36 | -2.2 (-5.4 to 1.0), p=0.17 | -1.3 (-4.7 to 2.1), p=0.44 | -2.9 (-6.0 to 0.15), p=0.07 |
| JADAD (total) |  | | | |
| Intention to treat | -0.7 (-4.9 to 3.6), p=0.74 |  | | |
| Adequate Sequence Generation | 3.7 (0.51 to 6.9) p=0.03 |  | | |
| Allocation Concealment | 3.5 (0.2 to 6.7) p=0.04 |  | | |
| Blinding | 3.9 (-0.9 to 8.8) p=0.10 |  | | |
| Incomplete outcome | -0.03 (-3.7 to 3.6) p=0.98 |  | | |
| Free of selective reporting | None were free of selective reporting | None were free of selective reporting | None were free of selective reporting | None were free of selective reporting |
| Free of other bias | None were free of other bias | None were free of other bias | None were free of other bias | None were free of other bias |
| Industry Sponsorship | -2.2 (-4.2 to -0.08), p=0.04 |  | | |
| Average Age | 0.42 (0.23 to 0.63), p<0.0005 |  | | |
| Drop-outs (%) | 0.08 (-0.06 to 0.22), p=0.27 |  | | |
| Study duration | -0.17 (-0.28 to -0.07), p=0.002 |  | | |
| Required NIH CPSI cut-off score | 4.3 (1.2 to 7.4), p=0.008 |  | | |
| Required subjective symptoms | 9.5 (2.3 to 16.8), p=0.01 |  | | |
| Required symptoms lasting longer than X months |  | | | |
| Required previous treatment with alpha-blocker | 1.5 (-4.7 to 7.7), p=0.61 |  | | |
| Alpha-Blockers | | | | |
| Publication bias | -5.5 (-13.6 to 2.7), p=0.15 | -2.0 (-6.4 to 2.4), p=0.31 | -3.7 (-7.0 to 0.42), p=0.08 | -2.5 (-5.9 to 0.8), p=0.11 |
| JADAD (total) | -8.2 (-16.2 to -0.19) p=0.34 |  | | |
| Intention to treat | 0.13 (-7.6 to 7.9), p=0.97 |  | | |
| Adequate Sequence Generation | 4.5 (-3.6 to 12.6) p=0.21 |  | | |
| Allocation Concealment | 1.13 ( -6.5 to 8.8) p=0.72 |  | | |
| Blinding | -1.9 (-11.8 to 8.0), p=0.64 |  | | |
| Incomplete outcome | -1.1 (-8.8 to 6.7), p=0.74 |  | | |
| Free of selective reporting | None were free of selective reporting | None were free of selective reporting | None were free of selective reporting | None were free of selective reporting |
| Free of other bias | None were free of other bias | None were free of other bias | None were free of other bias | None were free of other bias |
| Industry Sponsorship | -2.6 (-7.3 to 2.1), p=0.22 |  | | |
