# Supplementary material for: Therapeutic Intervention for Chronic Prostatitis/Chronic Pelvic Pain Syndrome (CP/CPPS): A Systematic Review and Meta-Analysis
Source: PLoS One. 2012 Aug 1;7(8):e41941. doi: 10.1371/journal.pone.0041941 (PMC3411608; doi:10.1371/journal.pone.0041941)
Supplement: Table S9 — Comparison of Current Classic (Pair-Wise) Meta-analysis and the 2011 Anothaisintawee et al. Network Meta-Analysis. (DOCX) [file pone.0041941.s011.docx]

**Table S9. Comparison of Current Classic (Pair-Wise) Meta-analysis and the 2011 Anothaisintawee *et al.* Network Meta-Analysis**

| **Criterion*** | **Current Pair-Wise Meta-analysis** | **Anothaisintawee *et al.,* 2011** | **Comment** |
| --- | --- | --- | --- |
| Well-formulated, clearly defined, answerable research  question | Yes | Yes | None |
| Patient population | More rigorously defined based on three levels of sensitivity of the definition of CP/CPPS | General definition of CP/CPPS | Blending patients from three levels of definition of CP/CPPS and treating them as a single group might “affected the validity of findings from a network meta-analysis” * |
| Interventions | Pharmacologic plus interventional studies | Limited exclusively to pharmacologic-only studies | Limiting analysis to pharmacologic approaches only might affect “the validity of findings from a network meta-analysis” |
| Comparisons | Pair-wise only | Pair-wise and network | Network meta-analysis offers the advantage of analyzing direct and indirect comparisons |
| Outcomes | NIH-CPSI-based | NIH-CPSI and other (non-CPPS-specific) questionnaires | NIH-CPSI is the quality standard for diagnosing CP/CPPS and ensures a more rigorous assessment of the outcomes |
| Study Selection | *De novo* original search strategy developed by librarian and original rigorous double-blind adjudication strategy | Traditional search strategy | Our strategy provides a more rigorous and reproducible way for avoiding selection and publication bias. |
| Languages | English, Chinese, Korean and Russian | English only | Our multilingual search strategy provides a more rigorous and reproducible way for avoiding language bias |
| Assessment of Risk of Bias | Only one pooled estimate of treatment effect (e.g. alpha-blockers), risk of bias - LOW | More than one pooled estimate, risk of bias - HIGH | Risk of bias varies across studies in the network meta-analysis (see Jadad and Cochrane Tables) which might affect the overall assessment of trials |
| Quantitative evidence synthesis |  | | |
| Heterogeneity (clinical) | Addressed by examining different levels of sensitivity of definition of CP/CPPS | Not addressed in network meta-analysis |  |
| Inconsistency | No inconsistency: Analysis limited to direct (pair-wise) comparisons only | Discrepancy between direct comparisons (e.g. lack of efficacy of individual medication groups) vs. indirect comparisons (efficacy of combination of antibiotics plus alpha blockers) |  |
| Bias | Addressed through sensitivity analyses | Not addressed | Might affect the interpretation of network meta-analyses since they might operate at different levels (e.g. publication bias, language bias). See Salanti et al., 2008** |

* Criteria based on Li T**,** Puhan MA, Vedula SS, Singh S, Dickersin K; Ad Hoc Network Meta-analysis Methods Meeting Working Group. *BMC Med.* 2011 Jun 27;9:79. PMID: 21707969

** Salanti *et al.* Evaluation of networks of randomized trials. *Stat Methods Med Res*. 2008 Jun;17(3):279-301. PMID: 17925316
